# Complex Perinatal Syndromes Affecting Early Human Growth and Development: Issues to Consider to Understand Their Aetiology and Postnatal Effects

**DOI:** 10.3389/fnins.2022.856886

**Published:** 2022-04-18

**Authors:** Roberto Frenquelli, Marc Ratcliff, Jimena Villar de Onis, Michelle Fernandes, Fernando C. Barros, Jane E. Hirst, Aris T. Papageorghiou, Stephen H. Kennedy, Jose Villar

**Affiliations:** ^1^Master Program in Child Psychoanalysis and Neuropsychological, Developmental Psychology Unit, Faculty of Psychology, National University of Rosario, Rosario, Argentina; ^2^Faculty of Psychology and Educational Science, Centre Jean Piaget, University of Geneva, Geneva, Switzerland; ^3^Oxford Maternal & Perinatal Health Institute, Green Templeton College, University of Oxford, Oxford, United Kingdom; ^4^Geneva Foundation for Medical Education and Research, Geneva, Switzerland; ^5^MRC Lifecourse Epidemiology Centre, Human Development and Health Academic Unit, Department of Paediatrics, Faculty of Medicine, University of Southampton, Southampton, United Kingdom; ^6^Nuffield Department of Women’s & Reproductive Health, University of Oxford, Oxford, United Kingdom; ^7^Post Graduate Course on Health in the Vital Cycle, Universidade Católica de Pelotas, Pelotas, Brazil

**Keywords:** complex perinatal syndromes, human growth and development, preterm birth, extra-uterine growth restriction, intra-uterine growth restriction, pregnancy and childhood

## Abstract

Complex perinatal syndromes (CPS) affecting pregnancy and childhood, such as preterm birth, and intra- and extra-uterine growth restriction, have multiple, diverse contexts of complexity and interaction that determine the short- and long-term growth, health and development of all human beings. Early in life, genetically-guided somatic and cerebral development occurs alongside a psychism “*in statu nascendi*,” with the neural structures subjected to the effects of the intra- and extra-uterine environments in preparation for optimal postnatal functioning. Different trajectories of fetal cranial and abdominal growth have been identified before 25 weeks’ gestation, tracking differential growth and neurodevelopment at 2 years of age. Similarly, critical time-windows exist in the first 5–8 months of postnatal life because of interactions between the newborn and their environment, mother/care-givers and feeding practices. Understanding these complex relational processes requires abandoning classical, linear and mechanistic interpretations that are placed in rigid, artificial biological silos. Instead, we need to conduct longitudinal, interdisciplinary research and integrate the resulting new knowledge into clinical practice. An ecological-systemic approach is required to understand early human growth and development, based on a dynamic multidimensional process from the molecular or genomic level to the socio-economic-environmental context. For this, we need theoretical and methodological tools that permit a global understanding of CPS, delineating temporal trajectories and their conditioning factors, updated by the incorporation of new scientific discoveries. The potential to optimize human growth and development across chronological age and geographical locations – by implementing interventions or “treatments” during periods of greatest instability or vulnerability – should be recognized. Hence, it is imperative to take a holistic view of reproductive and perinatal issues, acknowledging at all levels the complexity and interactions of CPS and their sensitive periods, laying the foundations for further improvements in growth and development of populations, to maximize global human potential. We discuss here conceptual issues that should be considered for the development and implementation of such a strategy aimed at addressing the perinatal health problems of the new millenium.

## Introduction

“the false order that hides the *complexity*”

paraphrased from *Hopscotch*, J. Cortazar 1963

Understanding complex perinatal syndromes (CPS), so as to improve health outcomes, depends in part on our ability to assess and absorb the near exponential growth of knowledge that characterizes the 21st Century. In this opinion piece, we focus on preterm birth (PTB) and fetal growth restriction (FGR) as important, closely interrelated, CPS with major public health implications. We aim to demonstrate that the production of research results and assimilation of knowledge on growth, development and overall health, requires an interdisciplinary approach and an acceptance that environmental, cultural and socio-economic factors during both intra-uterine life and at least the first 2 years of postnatal life must be incorporated into any conceptual framework.

At present, at least three obstacles hamper how research results are used to improve the health of a population. The first relates to the various ways that results are quantified. In general, new studies are compared with existing knowledge using standardized strategies for locating, extracting and summarizing effectiveness. In the health sciences, the widespread use of systematic reviews and meta-analyses has transformed the process of summarizing knowledge, through the identification of relevant studies with as little selection bias as possible, and the quantification of treatment effectiveness and risk factor associations. We have implemented this strategy, at both individual and institutional level, with considerable conceptual and practical benefits during the implementation of research projects (Villar et al., 1995; Khan-Neelofur et al., 1998; Mignini et al., 2006; Conde-Agudelo et al., 2018). Unfortunately, exercises to synthesize and compare research results in many of the ‘basic sciences” and some fields of social sciences and education have not yet reached the same level of sophistication.

The second obstacle, no less significant in the search for a comprehensive understanding of CPS, relates to whether and how research from complementary clinical specialties is synthesized to achieve conclusions that are holistic and interdisciplinary, rather than fragmentary and specialty-biased. A classic way of representing this obstacle is the well-known analogy of blindfolded people touching an elephant with their hands to identify what is being explored. Each person (or separate clinical specialist) describes a different part of the elephant’s body: as expected, each arrives at a detailed and certain description of their part, yet a fragmentary conclusion, without accessing the animal’s totality.

Thus, the integration of independently obtained, but complementary, biological and developmental knowledge, is an ongoing challenge, especially in PTB and FGR research, that requires interdisciplinary action. In addition to conceptual issues, there are a number of important practical problems for research integration, such as different units of measurement, formulation of scientific questions, multidisciplinary assembly of data and statistical analysis strategies. All these challenges persist, despite the availability of modern computer packages, specifically in the analysis of intermediate factors that include biological, social and cultural variables as well as the possibility of *in silico* simulations of complex biological systems (Francois and Hakim, 2004).

Furthermore, integrating variables from diverse disciplines with different precision and units of analysis is difficult, as in the case of genome-wide association studies (GWAS) with clinical outcomes in perinatal medicine. For example, Horikoshi et al. (2016) reported that 62 distinct GWAS signals at 59 autosomal loci explained only 2% (S.E. 1.1%), of the total variance of birth weight in term newborns. Despite the laborious and expensive process of undertaking a GWAS in thousands of samples, the main outcome variable in this report was “self-reported birth weight” from only one sample representing just over 1/3 of the total study population. Self-reported birth weight by adults is notoriously prone to error and bias as it invariably requires the subject’s recall of what their mother recalled. Similarly, bias in the actual estimation of gestational age at birth is also likely. In the same GWAS study, adjustment by gestational age was only undertaken “where available” and the births occurred many years before accurate gestational age assessment, using ultrasound, was in clinical use. This is important as gestational age is the single greatest predictor of birth weight and varies considerably across ‘term,’ i.e., 37–42 weeks’ gestation: in boys, mean birth weight is 2.8 kg at 37 weeks and 3.7 kg at 42 weeks’ gestation (Villar et al., 2014).

The third obstacle relates to the tendency to study CPS by separating them into discrete clinical entities according to the classical descriptive approach, similar to anatomical dissection. This led to the assumption that what is presented in its totality must be studied fragmentarily to be fully understood. Such methodological constraint, although valid in research exploring very technical questions, often confounds the conceptualization of a global problem, resulting in epistemological reductionism.

The CPS are typical conditions that should be seen from the paradigm of complexity,(Morin, 2015) which implies bringing into play multiple, diverse contexts of broad amplitude and interaction, avoiding linear, mechanistic interpretations. This would provide a comprehensive view of the health-disease continuum, from the molecular to the corporeal, based on research strategies that reflect real life as closely as possible (pragmatic clinical research). Examples are the differences in the magnitude of effect of clinical regimens or treatments for hypertension or diabetes, or for the prevention of PTB, when evaluated in randomized clinical trials (RCTs) under extremely controlled settings rather than in pragmatic conditions closer to everyday clinical practice.

We strongly believe that an interdisciplinary conceptual basis is required to integrate all knowledge relating to the two main perinatal CPS, PTB and FGR, that manifest clinically during pregnancy and/or the neonatal period but have consequences projecting into early childhood. Such integration is even more important when it is appreciated that these outcomes depend greatly on the mother’s pre-conceptual state but also have long-lasting effects throughout life, i.e., they are both outcomes of maternal conditions and risk factors for childhood morbidity.

Below we: (1) provide a definition of CPS incorporating their postnatal complexity, using PTB and FGR as examples; (2) compare the similarities and differences across populations that make it easier to understand the diversity of PTB and FGR, and their associated critical periods during pregnancy and early childhood, and (3) present specific issues relating to the long-term effects and complexity of human growth and development.

## Defining Complex Perinatal Syndromes

There are several ways of interpreting the word *complex* in the context of understanding the life-time risks and consequences that CPS impose on the developing child – PTB, FGR and stillbirth being the most relevant. We use the adjective *complex* to express the complexity of the interactions that constitute each syndrome, which are currently poorly understood because they are not approached as a *totality*, a whole. Of course, *complex* implies neither completeness nor confusing; rather integration and antireductionism.

Furthermore, we take *complex* to be synonymous with multifactoriality, to indicate that major perinatal conditions never depend on a single factor, but always on the *interaction* of factors. Finally, *complex* is used, following the Systemic-Ecological Paradigm of Health, to reinforce the fact that the various causal or risk factors do not pertain to a single clinical or scientific discipline, which calls for an interdisciplinary approach.

The approach to perinatal syndromes also needs to bear in mind that: (1) PTB and FGR are defined, as opposed to most conditions in medicine, by a time mark, nametions, at various levels of biological complexity, can be represented as trajectories of growth anly gestational age, that supersedes symptoms, signs, and other clinical, nutritional or laboratory characteristics; (2) their complexity is caused by a combination of pregnancy-related conditions, socio-economic, lifestyle factors, the environment and non-Mendelian genetics. These interactions are not completely understood or quantified, which makes it difficult to develop adequate preventive or interventional measures, and (3) after birth, PTB and FGR newborns possess phenotypic characteristics that influence their health, growth and development, and chances of developing a range of diseases in later life.

Therefore, in this paper, we propose merging causal aetiological factors (*complex*) to postnatal morbidity, growth and development (*syndrome*) and claim that, because of their aetiological diversity and different disease phenotypes, perinatal conditions such as PTB, FGR and stillbirth are both syndromes and complex diseases. This reinforces the difficulty in separating syndromes from diseases, so often confronted in clinical practice. Finally, to add another level of complexity to the understanding of CPS, there are competing aetiological characteristics and risk factors among these syndromes.

## Preterm Birth: A Paradigm of Complex Perinatal Syndromes

The PTB is defined in purely temporal terms as birth at less than 37 weeks’ gestation. It affects approximately 10% of all births and is the leading cause of perinatal morbidity and mortality worldwide, with long-lasting deleterious effects on neurodevelopment and cardiometabolic health the earlier birth occurs. We have argued, however, that the current definition is inadequate to identify the main mechanisms leading to PTB (Villar et al., 2021b).

A more productive approach is to recognize that PTB is a syndrome and seek common clinical patterns then define novel phenotypes associated with differential risks of perinatal morbidity and mortality (Villar et al., 2012). This new phenotypic classification system improves on existing terminology because it relies more on characterizing aetiology as opposed to merely describing the mode of delivery and associated symptoms/clinical features. However, the system could also be an oversimplification: although a single clinical condition dominates most phenotypes aetiologically, there may be significant heterogeneity and interactions within each category. Hence, what is further required is to investigate whether common underlying factors exist within these phenotypes, i.e., whether there are genetic, epigenetic, metabolomic or proteomic signatures with interactive effects that may uncover novel therapeutic targets. A similar approach is justified for FGR as it also has overlapping patterns of risk factors and interactions that increase the risk of adverse outcomes.

The approach is based on concepts described above, i.e., the notion of causality is not only centered on slicing up the reality presented by the facts. It is about assuming multiple causality in a dynamic process, involving the interaction of a vast number of biological mechanisms, the environment and functional capacities. The PTB phenotypes we have identified, based on aetiological factors, are an example of this dynamic interconnectivity.

**FIGURE 1:**
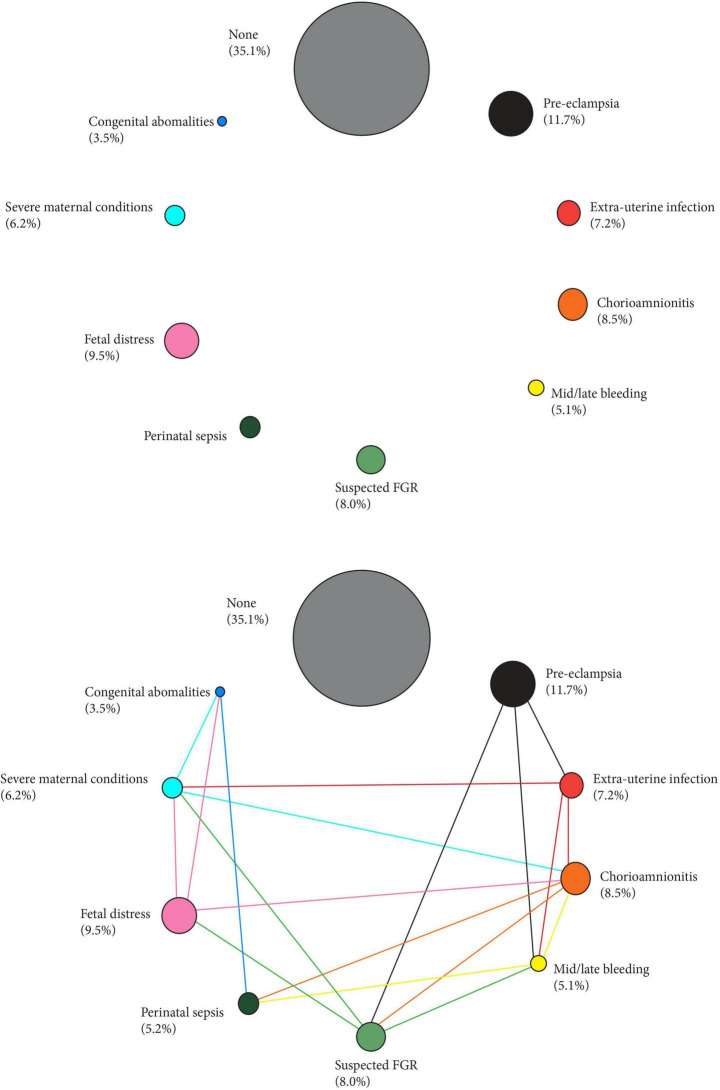
Preterm phenotypes and the complex interaction amongst their aetiological factors. Size of the circles represent the prevalence of the phenotype (%) in a recent multi-country population (Villar et al., 2021b). Links across phenotypes adapted from Barros et al. (2015).

Figure 1A shows the proportion of each phenotype to the PTB total, with the different sizes of the circles, corresponding to each phenotype, based on their proportional distribution in a multicountry population we recently studied (Villar et al., 2021b). Figure 1B shows how all the aetiologically-linked conditions for PTB are interrelated (see also the animated version) (Barros et al., 2015).

**FIGURE 2:**
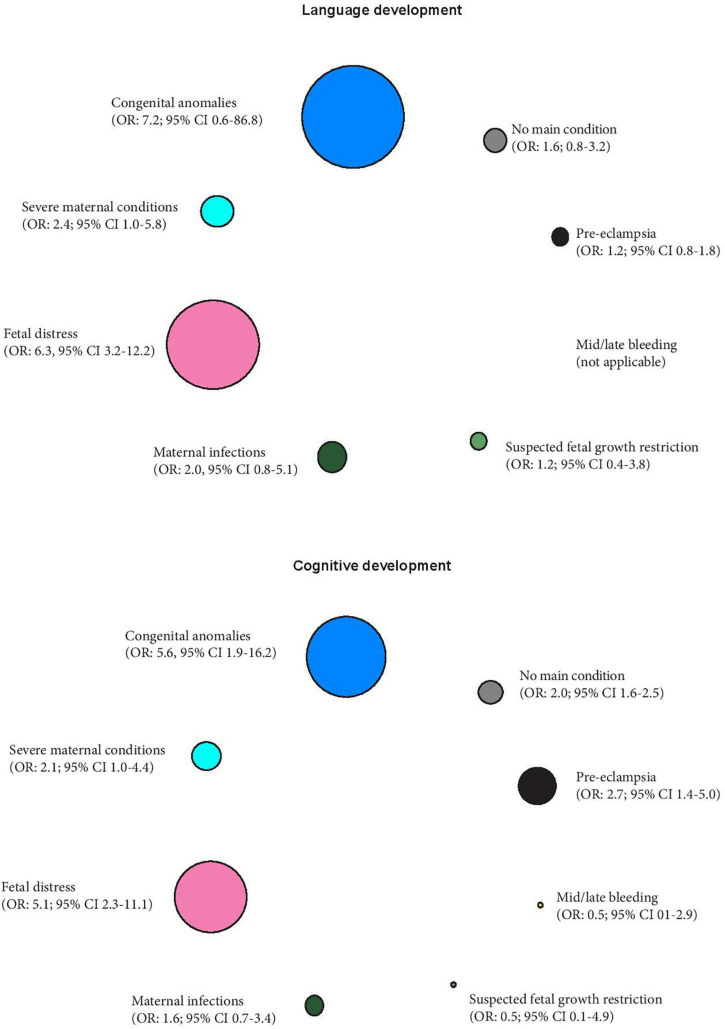
Preterm phenotypes and the association with early childhood language and cognitive development expressed as odds ratios (OR) and 95% confidence intervals, using term newborns as the reference group (Villar et al., 2021b). The different sizes of the circles represent the strength of the associations.

Furthermore, we have quantified the strength of the association between these PTB phenotypes and two early childhood developmental outcomes (language development and cognitive development) also obtained from our previous work (expressed as odds ratios; 95% confidence intervals), using the term newborns as the reference group (Villar et al., 2021b). The differential effects of these phenotypes on the two developmental outcomes are presented in Figure 2, with the sizes of the circles representing the strength of the associations.

To add another level of complexity, there is debate about whether the mode of delivery influences neurodevelopment outcomes in PTB newborns (Obican et al., 2015). On one hand, there is strong evidence that unnecessarily high Cesarean section rates are associated, independently of the risk profile of the population served, with increased rates of adverse maternal and neonatal outcomes, including PTB (Villar et al., 2006, 2007). Cesarean deliveries have also been associated with low levels of birth signaling hormones and less diverse microbial colonization linked to disruptions in the developmental programing of the neonatal brain with subsequent long-term consequences on neurodevelopment (Kenkel, 2021). On the other hand, it has been suggested that Cesarean deliveries have a “theoretical advantage” in premature infants due to the avoidance of prolonged labor and less traumatic birth (Obican et al., 2015). This is a complex literature, and cannot be discussed in detail here; however, most RCTs and cohort studies that controlled for possible confounding factors have found no associations between mode of delivery and neurodevelopmental outcomes in PTB infants (Bahl et al., 2007; Zhu et al., 2014; Obican et al., 2015; Chimenea et al., 2022). It is likely that the observed relationships in non-randomized studies were confounded by the clinical indication for Cesarean delivery rather than to mode of delivery.

## Fetal Growth Restriction: Aetiological Factors, the Environment and Outcomes for a Complex Perinatal Syndrome

The interactions, at various levels of biological complexity, can be represented as trajectories of growth and development across both gestational and postnatal ages, that express “patterns” or “patterns that connect.” These are particular types of relationship, i.e., intelligible, somewhat discrete patterns that, at the same time, allow totalities to be captured in a temporal context. Where multiple/inter/poly-retroactions operate, we could even speak of “patterns of patterns” or “meta-patterns,” where these differences always exist within the structure of the whole, and are integrated and delineated in the trajectories obtained (Bateson, 1972, 2002).

Of interest, is the identification in these trajectories and meta-patterns, periods that could indicate crucial moments in development, as demonstrated for example by the relationship between: (a) infant late weaning and maternal closeness and (b) cognition and behavior at 2 years of age (Villar et al., 2020). The biological foundation is provided by the evidence that a large number of developmental and maturational processes occur simultaneously and synergistically with the timelines for synaptogenesis and pruning varying between different brain regions. It is possible, therefore, that disruptions at different windows of sensitivity may confer differential delays in neurodevelopment.

Further biological evidence supporting the link between vulnerable periods of brain plasticity and disorders of development and early life adversity, is provided by recent studies of the role of cellular timing mechanisms (e.g., chromatin remodeling, circadian clocks, redox balance, and neuronal oscillations) and neuromodulation involving the balance between the maturation of neocortical inhibitory processes (e.g., GABAergeic, glutamatergic and parvalbumin-positive systems and excitatory synaptic processes) in the maturation of developing nervous systems. This is a fascinating, yet extensive literature that cannot be properly discussed here but has been recently covered by Patton et al. (2019) and Reh et al. (2020).

We have constructed phenotypes within the CPS, by incorporating aetiological components, contributing to a better understanding of the exposome, (Wild, 2005) characterized by the effects on possible multiple causal exposures such as environmental factors, endogenous metabolic processes, nutrition and pregnancy-related conditions. Then, as an expression of these numerous encounters that epigenetics helps to explain, we come close to a comprehensive concept, that we call “Ecological-systemic approach to Perinatal Health,” with multiple levels of measurement up to the fields of genetics and omics.

We propose to think of “texts” in different “contexts.” It is evident that, if the human body has evolved complexly over hundreds of thousands of years, then one must first understand this complexity in order to understand its totality. Additionally, biological systems develop, during early life, through a relationship with the environment (intra- and extra-uterine), albeit with wide-ranging intra-individual variation in response to risk and protective factors (Fernandes et al., 2018). In the context of FGR and other CPS, it is imperative to include an understanding of how environmental exposures interact with genotypes and phenotypes to shape human growth and development differentially.

Moreover, as we describe below, it is vitally important to appreciate that all humans belong to the same species and that, in terms of growth and development, within-group variability is greater *than* the variability among different populations if the living conditions required to achieve the genetic potential of the human species are met.

However, as being born too early and/or too small can have potentially deleterious effects on subsequent growth and neurodevelopment, we believe the complexity of FGR (as well as PTB) and their potential consequences, cannot be comprehended without considering postnatal events, at least until the critical period of 2 years of age. The assessment of these interactions on life-course trajectories requires longer follow-up periods but will, for the most part, remain based on a detailed description of the specific organs and functions affected during the fetal and neonatal period.

## Complexity in the Origins, Complexity in the Outcomes: The Case of Postnatal Growth and Development

What do we understand by the term ‘postnatal events’? We consider the physiological bases of growth and development to be a means of achieving the purpose of living – which is nothing other than “continuing to be” – a conjugation of being and becoming, in which various functional structures, including the psyche, are emergent properties of organized matter (Laborit, 1976).

For example, we can ask ourselves to what extent the grasping reflex is part of the physiological domain or of the incipient psychological domain. From this perspective, as soon as one considers the simplest developmental milestone, such a distinction is impossible. The same goes for the different smiles (reflex and social) and languages (analogical, iconic, and digital).

The function of the psyche is nothing other than a materially based disposition in pursuit of life – life that is always relational, contextual. Because the human environment, first and foremost, includes our fellow human beings. The faculty of grasping, once detached from the basic reflex automatism, is then intimately linked to the need for incessant interaction with fellow human beings. As such, a child holding hands with their parents expresses the need for emotional support, for attachment. This represents a temporal process with a passage from quantitative, individual actions, to qualitative creation of a complex series of links and connections with our social and emotional environments (Sabelli, 1989; Sabelli and Kauffman, 2005). We will follow the saying “survive first, live later,” through which we apply a formula where the priority is the basic needs for human survival, and from where the psychological aspects of life emerge progressively intertwined.

Development, including neuropsychological development, is always considered as an emerging property of organized matter, within the evolutionary scale, where the term emerging in no way implies a unilateral deployment of any of the elements involved in the development. All novelty is always being interactional. Before 25 weeks of intra-uterine life we are witnessing a psychism “*in statu nascendi*,” given that the neural structures have already laid their foundations (Frenquelli, 2005).

Psychic development is thus the result of a process in which there is a passage from quantity (cellular growth), to cranial and cerebral size, to quality (functional), all interacting and giving priority to physical growth with its nutritional properties in close relationship with the environment. Quality that is based on organized matter, with the differences that take place in growing structural levels, in the style of Russian dolls.

In the case of neuropsychological development, it is now acknowledged that while the substrate for susceptibility is neurobiological in origin, genetic susceptibility factors operate through neurobiological processes, with outcomes at any time point in the life course being additionally influenced by intervening intrinsic and extrinsic experiences (Fernandes et al., 2018).

## Human Growth and Development Within and Among Diverse Populations: From the Similar to the Different

It is important to conceptualize that across non-isolated populations, the variability in skeletal growth and neurodevelopment from the fetal period to childhood is *greater* within a geographical, religious, cultural group or population where conditions of health, nutrition, education and general environment are adequate, than among groups of individuals, living in similar conditions but in different geographical, religious, skin color or ethnic regions.

**FIGURE 3:**
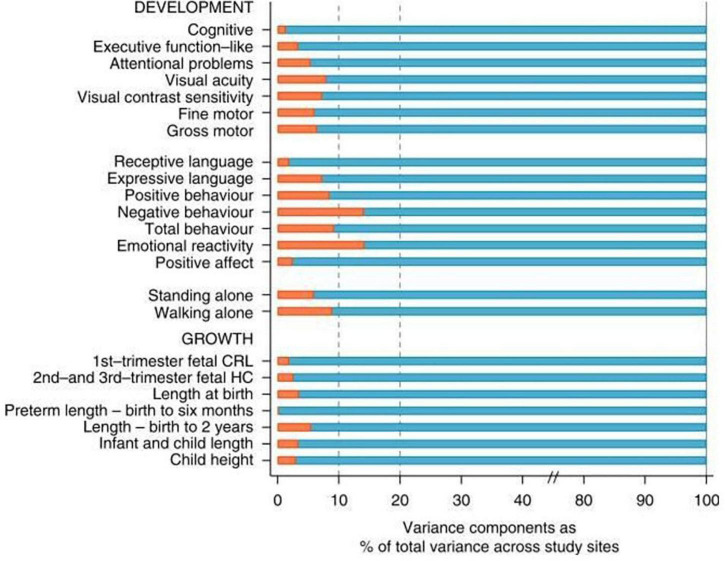
Variance components analysis of 16 neurodevelopmental domains evaluated in the present study **(Upper)** and variance components analysis of 7 measures of fetal, newborn, infant, and child growth **(Lower)**. Red bars are the % of total variance explained by between sites variability (Villar et al., 2019).

A quantification of these similarities in skeletal growth and development among varied populations has been produced by the INTERGROWTH-21st Project using analysis of variance (Villar et al., 2013). Figure 3 Villar et al. (2019) presents the percentage of the total variability that corresponds to the variability among geographically different populations when health, nutritional and educational conditions are adequate.

As can be seen, the percentage of the total variability (100%) that is due to differences among groups is never more than 10% of the total variability in these indicators of growth and neurodevelopment with the exception of negative behavior and emotional reactivity that marginally exceed 10%. In short, under adequate environmental conditions for growth, health and nutrition, geographically dispersed populations are more similar (*integrated*) than their individual members are to each other (Villar et al., 2019).

Supporting these clinical and epidemiological observations, there is good evidence that the overall genetic make-up of people from different ‘races’ is also similar and that at least 85% of genetic variation is accounted for by within-population inter-individual differences, not by differences among groups (Rosenberg et al., 2002). Specifically, common genetic variants only explained 15% of the variability in birth weight in the GWAS described above (Horikoshi et al., 2016), a proportion very similar to that shown for growth and development in Figure 3. Of relevance to this proposition, is the evidence from ovum donation studies, that birth weight is more closely related to the recipient’s body size than that of the donor (Brooks et al., 1995).

Furthermore, despite differences in the prevalence of disease-associated genetic variants across populations, the impact on the risk for common diseases is usually consistent across ‘racial’ groups (Ioannidis et al., 2004). Conversely, in a high-risk environment because of nutritional, health care, educational or socio-economic constraints, the prevalence of PTB and FGR is increased overall among groups and geographical areas.

**FIGURE 4:**
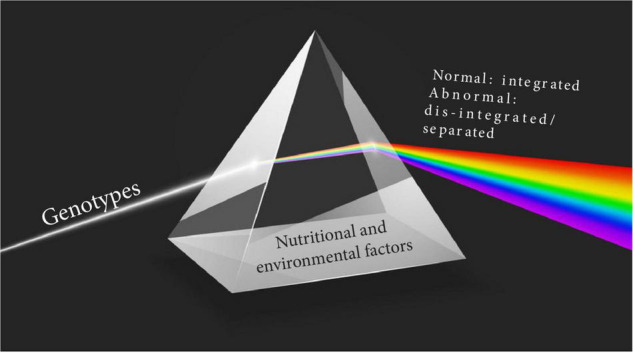
Schematic representation of the interaction between environmental factors and clinical phenotypes.

However, how the phenotypes express themselves clinically varies according to: (a) the prevalence, type and duration of specific risk or causal factors and (b) the characteristics of the individual mother–child dyad – all of which vary according to the socio-economic and geographical contexts. An example of the complexity of the situation and the effect of risk factors is seen in populations under “epidemiological transition” where the changing prevalence rates of obesity and malnutrition coexist according to the stage of transition. In other words, the “abnormal” is *disintegrated*, i.e., dissociated across populations as the prevalence of different insults, exposures or risk factors changes at a local level. These changes and their consequences constitute “exposotypes,” which complement the classical concept of CPS phenotypes by adding the external etiological component (Rattray et al., 2018), as we have recently described in relation to PTB (Villar et al., 2021b) (Figure 4).

**FIGURE 5:**
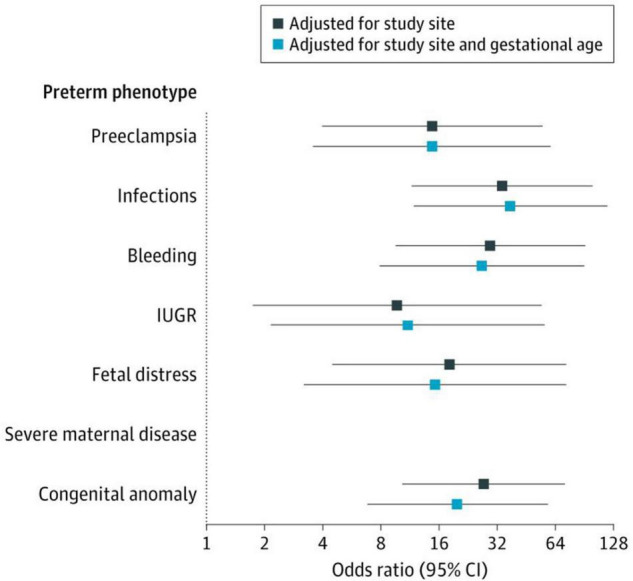
The Severe Neonatal Morbidity Index (SNMI) according to the eight preterm birth phenotypes. SNMI includes bronchopulmonary dysplasia, hypoxic-ischemic encephalopathy, sepsis, neonatal anemia (requiring transfusion), periventricular hemorrhage or leukomalacia, retinopathy of prematurity, necrotizing enterocolitis (Bell Stage 2 or higher), and patent ductus arteriosus (requiring pharmacological treatment or surgery). There were no cases of severe neonatal morbidity in newborns with the severe maternal disease phenotype. Odds ratios estimated in comparison with the preterm phenotype “No main condition detected”. The 95% Cls were based on robust SEs. IUGR refers to fetal growth restriction (Villar et al., 2021b).

Figure 5 shows, as an example, that the risk of severe neonatal morbidity varies for the different PTB phenotypes we have described, independently of geographical location and, most importantly, gestational age. This demonstrates that causal factors, independent of gestational age, determine an important component of the risk of subsequent morbidity and growth and development in childhood, i.e., intra-uterine life inflicts specific damage that affects postnatal life (Villar et al., 2021b), especially at critical periods during pregnancy.

## Critical Periods for Human Growth and Development

**FIGURE 6:**
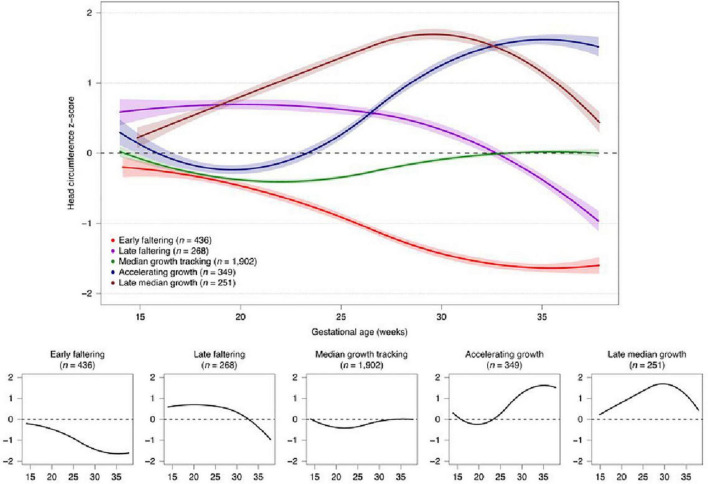
Fetal cranial growth trajectories in the INTERBIO-21st study (Villar et al., 2021a).

We have previously identified different trajectories of ultrasound-derived, fetal cranial growth that clearly diverge, in a similar manner to fetal skeletal growth, during the period prior to 25 weeks’ gestation (Figure 6) (Villar et al., 2021a).

Importantly, these cranial growth trajectories are associated with differential growth and development at 2 years of age (Villar et al., 2021a). Therefore, the fetal developmental “window” around 20–25 weeks’ gestation should be considered a critically sensitive period during and before which factors affecting the fetus have a profound effect, particularly on neurodevelopment. This effect could be related to the finding that the total number of life-time, neocortical, excitatory neurons is determined before 27 weeks’ gestation (Ernst et al., 2014).

The period of rapid development during the early second half of pregnancy that continues into early infancy is regulated by excitatory neurons and the cortical GABAergic system (Xu et al., 2011). This is relevant because γ-Aminobutyric acid (GABA) comprises >20% of all interneurons, (Raju et al., 2018) is the major inhibitory neurotransmitter in the mammalian central nervous system (Hachiya and Takashima, 2001; Xu et al., 2011) and GABAergic deficits have been associated with psychiatric conditions (Reynolds et al., 2004; Luscher et al., 2011; Coghlan et al., 2012; Edden et al., 2012) aetiologically linked to fetal undernutrition, viral infections, alcohol and cannabis (McNeil, 1991; Smiley et al., 2015; de Salas-Quiroga et al., 2020). Specifically, there is evidence that the period between 22 and 26 weeks’ gestation is a critical window for the development of GABAergic neurons in humans. Cellular lineages of neocortical GABAergic neurons show migration between 14 and 24 weeks’ gestation, (Letinic et al., 2002) and central and periventricular white matter GABAergic neuronal density significantly increases from early pregnancy to peak around term, with decreases thereafter (Xu et al., 2011). Markers of GABA transporter-1 (GAT-1) – the most widely distributed of the GABA transporters– are observed in the dentate gyrus of the human hippocampus as early as 21–22 weeks’ gestation and in the stratum lacunosum-molecule are at 26 weeks’ gestation (Hachiya and Takashima, 2001).

Similarly, data on neurodevelopmental outcomes in children born extremely preterm show that 24 weeks is the gestational age threshold for a significant change in rates of cerebral palsy, motor delays and vision and/or hearing impairments, while the threshold for a significant positive change in cognitive outcomes is birth before/after 25 weeks’ gestation. We acknowledge that PTB itself is another outcome rather than a cause and that sensory overstimulation and medical interventions may themselves interrupt developmental processes of these extremely preterm infants (Fernandes et al., 2021).

The notion of a critical (sensitive, unstable or vulnerable) period should also be viewed as a window of opportunity for improving health outcomes (Glover, 2011). Coined in the fields of ethology and neurophysiology, the term implies the existence of moments in the developmental trajectory (especially early), where highly significant effects (both favorable and unfavorable) can be produced. Learning to sing in birds is a classic zoological example, as adequate stimulation, in time and form, is essential for the development of this aptitude (Nottebohm et al., 1976).

These temporal windows are key moments for establishing the necessary neuronal aggregations that will constitute the support for emotional regulation, sensorimotor performance, and the representations required for language. The concept of special periods has attracted considerable attention in the field of early education and been promoted as an opportunity to reach higher levels of child development (Magistretti and Ansermet, 2010). Of course, damage that occurs during these periods may be corrected, either partially or totally, by compensatory mechanisms during childhood depending on the severity and nature of the insult.

The finding that fetal cranial growth trajectories are associated with different outcomes up to 2 years of age is also an example of how a part of the structural growth process, in this case indirectly reflecting brain growth, can manifest itself in functional aspects of development (cognitive, motor, language, and behavior scores) as a whole. We interpret these trajectories of fetal neurodevelopment as a broad, integrated, systemic process, requiring each individual component within this perspective to be understood. However, each organic element has its own specific temporal factor, e.g., cerebellar growth, (Rodriguez-Sibaja et al., 2021) that can contribute toward specific phenotypes vis à vis the overall developmental process.

## Preparation for Developing in Stages

The evidence for critical or sensitive periods, with effects expressed in later behavior, would seem to contradict the concept that preceding periods of baseline development must be completed before moving on to more complex developmental levels, as classically proposed by Piaget (1952). However, in our opinion, these two time-based characteristics of human development are in fact complementary rather than contradictory (Piaget, 1952). We have recently explored these issues in a prospective study of over 1,200 mother/child dyads (Villar et al., 2020). We have shown that longer breastfeeding and late weaning have a beneficial effect on gross motor development during early childhood, which facilitates more active, exploratory activities of the infant within their environment, i.e., the value of sensorimotor skills as the motor for cognition (Piaget, 1972, 1976).

Similarly, we have described a positive association between the introduction of semi-solids to the infant diet (partial weaning) after 6–7 months of age with the child’s visual capacity: the longer the breastfeeding, the better is vision at 2 years of age (Villar et al., 2020). This underlines the importance of visual capacity during the period when body movements begin to be coordinated.

In addition, we have observed that the total time exposed to formula feeding in this unique cohort of healthy, well-nourished infants across diverse geographical locations, adjusted for the age on initiation of formula use and a set of confounding variables, was systematically associated with lower scores in five of the eight neurodevelopmental domains/items. Such a negative association was not observed with the fine motor, early walking and vision outcome (Villar et al., 2020). It was in a mirror fashion, opposite to the positive effects observed for the indicators of breastfeeding. These results support a very large literature on this subject, including systematic reviews (Horta et al., 2015; Horta and de Lima, 2019) and narrative discussions (Krol and Grossmann, 2018) providing evidence about the benefits of human milk and breastfeeding with their nutrient and nursing roles, (Pang et al., 2020) particularly in less developed countries (Victora, 2000; Victora et al., 2016).

Thus, these data demonstrate that consistent, external, nutritional and affective support (breastfeeding) during a critical period of development, requiring the highest level of energy expenditure as well as near absolute dependance on external care (Pontzer et al., 2021), promotes a basic sensorimotor level, which in turn is required for reaching advanced levels of intelligence in childhood (Villar et al., 2020).

Furthermore, we have also explored the interaction between breastfeeding, motor development and more complex behaviors during early childhood and found a positive association between the duration of exclusive breastfeeding or the age of introduction of semi-solids to the infant’s diet with higher scores in markers of “emotional reactivity” and “attention problems,” all within normal values, without pathology (Villar et al., 2020).

We have explained these results based on the concept that the 2-year period of tantrums or rebellion against the mother – Spitz’s so-called “terrible 2 years” (Spitz, 1957) – should be considered a period requiring a degree of motor-emotional security to cope with the conflict of the mother’s demand and an incipient level of independence. For this “rebellion,” the child must have reached a sufficient degree of neuromotor maturation to run independently or climb stairs. We suggest that the (critical) extended period of breastfeeding helps this development either through a nutritional effect or by more secure attachment (linked to emotional regulation), or more likely because of an interaction between both factors (Bowlby, 1988).

It should clarified that the term “breastfeeding” includes “breast” as well as the game of gaze, positions adopted in the act of suckling and expressed maternal milk for very preterm infants. We specifically refer to “mother/care-givers,” denoting that the provision of infant care can be carried out indistinctly by different social actors, including the father. Actually, in a recent publication we have included the concept of “Gangs of mothers,” defining the “mother” as the main person feeding the child versus another person(s), including the father (Villar et al., 2020). We do not suggest that the mother has to be constantly present because care is provided currently in most societies, by a permutation of family members and care-givers. In fact, our recent results (Villar et al., 2020) do suggest that, during this multiphasic period of the first 8 months of life, there are inter-connected environmental factors and mental processes that substantially contribute to the psychological make-up of humans.

Finally, there may be another critical period around the 8th postnatal month, 2 months after the recommended period of weaning, in which there is a higher level of integration, i.e., “appearance of the will” including a degree of intentionality that separates the two movements of assimilation: a desire is integrated in the cycle of assimilation and accommodation (Piaget, 1952).

## From the Archaic to the Social and Back to the Archaic: The Neonatal Hand-Mouth Reflex “Reappearing” Later in Life?

The hand-mouth reflex, can be considered one of the first auto-organization processes where an extraordinary expansion from the mouth to the hand links nourishment to pleasure. The existence of a “mouth self” is often suggested, after Stern’s “buccal space” analyzed by Piaget (1952). This oral component has been described as the “rooting behavior complex” and includes the pressor movements of the infant’s hands on palmar stimulation, i.e., the hand “reflex” or rather the response, the grasp reflex. This complex scheme of activities will eventually be complemented by thumb sucking. It is real knowledge embodied in early memory patterns, indelible memory, which permeates all later life. Indeed, Piaget’s notion of scheme includes a recognition dimension.

This initial complex behavior, perhaps the earliest in humans, provides instant pleasure to the newborn associated with suckling, as well as access to nutrition. Therefore, the assimilation of objects to the mouth and the coordination of hand and mouth are essential components of early ego integration.

Today, young adults have adopted a permanent hand “reflex,” a grip stimulated by the continual handling of mobile phones. A variation of this position can be observed in a large number of people who speak on the phone as if they were being bottle fed (similar to eating a hot dog). Some young adolescents even adopt the breastfeeding position when lying on a sofa or bed at the same time as using their mobile phone. Can adopting this set of positions, which are not evidently necessary for mobile phone use as such, be related to a return to one of the most archaic of human reflexes and positions? Or this could be a manifestation of the proposed infantilisation of Western societies, made worse because young adults are facing widespread financial uncertainty, political crises and the pandemic?

This breastfeeding position associated with mobile phone use may further stimulate hunger and appetite associated with vestibular proprioceptive processes as it does when a newborn is in the breastfeeding position. All these are linked to the need for immediate connectivity, a kind of automatic and unreflective connectivity.

By presenting this hypothesis here we are not at all promoting an “anti-technology” stance. It is a call for attention to the establishment of certain habits and their effects on everyday gestures that insensibly infiltrate our way of life, but are based on the most archaic of human experience that re-surface, later in life, within new social interactions.

## The Complexity of Information Production, Organization and Interpretation

There are levels of “in-formation,” expressed in structures alongside disciplines, which exchange messages with each other in a permanent game in search of homeostasis – a form of regulation in the understanding of complex problems. There is also “circulating information,” which in turn links individuals to the environment. Living beings can only be conceived as thermodynamically and informationally open systems. There is a necessary dependence on the outside, although they always seek to comply with their own rules, self-organized, “self-poietic,” insofar as they produce themselves.

In this way, living beings “are molecular plots and interactions that produce themselves, specifying their own limits.” They are an accumulation of circuits that constantly draw interactive loops. It is noticeable that we permanently speak of circuits of sensation and action. Action as operational closure, which is first of all sustaining, balancing, but then gives rise to more and better achievements. There is an incessant interplay of levels from subatomic structures, to atomic structures, to molecular structures, to tissues, to organs, to systems up to the individual subject. And from the human being to the environment, which of course includes – primarily – other humans (Maturana Romesín and Varela, 2009).

The maternal-fetal unit is the typical case of this complexity at all levels from molecular to organ level; however, there is the added complexity of bi-directionality (mother to fetus, and fetus to mother) involving the placenta that acts as an intermediary level to regulate, filter and transport nutrients and oxygen as required and as available. Interestingly, the challenge is sometimes to identify the direction and objective of such biological processes, e.g., phosphatidylcholines acting as transporters or messengers according to their side chains.

Living beings endowed with a nervous system are dependent on the environment in which they live, but at the same time they are capable of forming their own structure, independently. It is in these contexts, in these incessant messages between the different levels of organization, that integration takes place over the course of time that involves the whole of development. A memory is configured in the process, through connections, whereby each text is inscribed in certain and specific contexts. It is necessary to submit them to permanent contrast and analysis. When we speak of memory, we refer to a trilogy that includes genetic, immunological and nervous memories (Kandel, 2007).

The widely cited comparison between the best of the bees and the worst of the architects could apply to fetal growth monitoring and gestational age assessment that are the keys to diagnosing FGR. We have evaluated the introduction of artificial intelligence or machine learning to clinical practice: is it closer to human operators, better or just “a better bee” that uses human brains for programing, whilst avoiding human bias? We have estimated that ultrasound equations used to estimate gestational age early in pregnancy (a key tool for the diagnosis of the PTB syndrome) implemented by expert operators have error in the measures with clinical implications (Papageorghiou et al., 2014). A more advanced machine learning equation has reduced such errors to around 3 days (Fung et al., 2020) with implications for the diagnosis of PTB – a clinical syndrome that is currently defined exclusively on a temporal basis (Marx et al., 1890).

## Diversity of Functions During the Perinatal Period

The mouth is the seat of sucking, which is basic for early feeding. But it is also the seat of early communication including facial expressions, e.g., smiling and frowning, as it is for pre-speech and speech (Parsons et al., 2010; Darwin, 2015). One and the same place opens up a range of properties and their meanings, which cannot be understood without realizing that they are intimately intertwined. It is a dynamic structural process, where the subject actively co-constructs itself in successive encounters with the environment, following unavoidable steps that mark its evolutionary character.

The mouth therefore constitutes an environment in which numerous functions are rooted, which come together in a joint, successive, or alternating manner. This fact, as simple as it is complex and forceful, gives us an idea of the way in which our physiology is embodied, as a true knowledge, always linked, expressed behaviorally – a knowledge that is processed in different ways, both in the being and in the becoming of the subject (Bleichmar et al., 1994). This multitude of oral reflexes, plus their relationship with the manual, visual-spatial and proprioceptive-motor, always intertwined with emotion, make up an unavoidable example of the multiplicity that we sustain.

Similarly, the breast has a number of diverse functions. We have explored the multiphasic, fundamentally important first 6–8 months of a newborn’s life, when maternal interactions, environmental factors and early mental processes can have different maturational influences. It is during this short period of human life that the initiation of “object relations,” dependency, dependence and attachment with the mother are present. As Freud recognized, “At the woman’s breast love and hunger meet” (Freud and Brill, 1913).

The brain is at this stage an experience-dependent, as well as experience-expectant, organ – waiting for significant others to organize itself from the first steps of its ontogeny, from the first neuronal paths, from the first traces of memory (Wiener, 1961). For example, a physiological “decision-making” process has been postulated in fetuses with FGR at the level of placental flow that reduces circulation to the fetal liver, whenever nutrients and oxygenation are limited, so as to protect the brain. How effective this process is in deciding which organ is the winner and how much memory these organs have to cause a deleterious effect during adulthood in various neonatal contexts is an area of ongoing study (Aitken and Trevarthen, 1997; Severi et al., 2000).

A true inter-phase between our inner and outer selves, our brain (along with our skin, of similar embryonic origin, and the largest of our senses) is becoming the seat of a plot where reality, ungraspable, is refracted in the prism of neural networks (von Foerster, 1992). A series of sensorimotor processes, in the heat of the emotions, sculpt our singular identity – never the product of a copy – with instruction from the environment. This always operates only by disturbing the dynamics of internal states. Thus, a social history is always incarnated in the singular human body (Raitzin de Vidal, 1988).

## Complexity and Diversity: The Dynamic Process of Exposures, Outcomes and Their Interactions

Our nervous system, with those materials coming from externalities, is self-organizing. That is to say, it is far removed from the simple stimulus-response model. Seen from the paradigm of complexity, the notion of subject implies the consideration of a set of relations that cross the levels of organization. And from the individual to the other humans that constitute his or her social environment.

Basic and acquired needs pass through an inevitable diversity, amalgamating in a constant way in poly-, inter-, and retro-actions, fulfilling the three postulates of complexity: dialogicity (interaction of opposites), hologram (the part is in the whole and the whole in the part) and recursivity (producer-product indistinction).

We admit the existence of circular chains of causation, where the cause-effect sequence vanishes. Mechanistic linear determinism is superseded by an undivided circular trace. Cause and effect become admissible notions as mere arbitrary punctuations. Loops of action and feedback are delineated, which cybernetics has come to conceptualize in depth (Morin, 1992). Living beings endowed with a nervous system are capable of producing new assemblies, novelties. The associative capacity of human beings, source of imagination, creation of doubts and decision-making, is prepared from the genetic template during intrauterine life, expectant of the environmental encounters that are essential for the achievement of their objectives and performances.

## Conclusion

We strongly support the concept of a continuing interaction between growth and development from conception to childhood and from the molecular to the systemic level. CPS, as they present during very early periods in human life, represent a typical case of these interactions and complexities, which are even more complex as their pathological manifestations influence adult life and health, and the complex includes the simple and the integrated. It is within this conceptual framework that we have attempted to frame our CPS research.

A fact yet to be fully conceptualized is the relationship between normal and pathological growth and development and the level of complexity at which it should be studied. What additional insights will genetic and omic studies provide? Genetics has clear definitions and “reference” patterns; others are more difficult to interpret in the context of specific clinical conditions since, in the end, all metabolic and physiological, including mental, processes must be implemented by basic functions at the sub-atomic level.

We further argue that the most efficient way to deepen knowledge of PTB and FGR and their associated adult complex clinical syndromes, such as cardiovascular disease and metabolic syndrome, as well as their variations over time, is a permanent contrast and analysis of the different biological levels and their possible causes and associated factors. Recognizing their complexity, the analysis of CPS should approach neither the complicated nor the confusing; it should be meticulous but sufficiently flexible to access the whole, whilst at the same time including and surpassing it.

We propose a systemic, dynamic and multidimensional perception of growth and development starting at the molecular/genomic level that allows an understanding of all the inter-related CPS, delineating temporal trajectories and their conditioning factors, but one that permits the incorporation of new, rigorously evaluated, reproducible scientific evidence (Raitzin de Vidal, 1988).

Focusing on potential growth, neurodevelopment and temporal curves must leave room, especially during periods of greatest instability or vulnerability, for the generation of new phenotypes and prevention of impairment, and for structural social changes that build human capital. Such patterns are the threads of life processes that alternate between health and disease.

The potential universality of human growth and development is affected by a rhizomatic multi-causality with the temporality of biological phenomena and their interactions with the environment, manifesting as different fetal and infant phenotypes and exposotypes. Thus, substantial progress in understanding FGR and PTB as the main CPS requires a holistic view of reproductive and perinatal issues, whilst constantly acknowledging the enormous potential of the human species within its individual variability.

**FIGURE 7:**
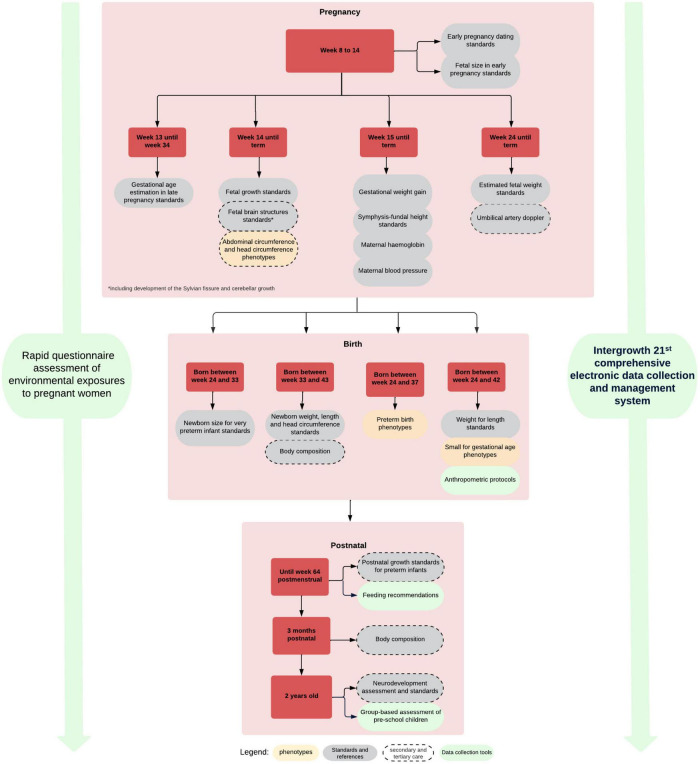
INTERGROWTH-21st growth, developmental standards, clinical and data collection tools.

We have demonstrated the similarities among populations in growth and development when health, nutritional and environmental conditions are adequate including the concept of relative a temporality of growth and development standards (Villar et al., 2014). We have produced, based on this evidence, a set of integrated international tools and standards (Figure 7) for monitoring growth and development from early pregnancy to childhood that are being implemented worldwide (Papageorghiou et al., 2018). These tools and standards are complemented by a comprehensive data gathering system including pathological determinants, socio-economic and nutritional status, as well as development during pregnancy and after birth (Chatfield et al., 2013; Cheikh Ismail et al., 2013, 2016; Eskenazi et al., 2013; Fernandes et al., 2014; Slim et al., 2018).

Conversely, pathological conditions such as CPS cannot be presented and described as a fixed, permanent situation vis à vis the underlying populations, despite the atemporal nature of international growth standards. The prevalence of risk or causal factors and their interaction with the health care system responsible for the diagnoses (with eventual treatments) and the environmental conditions in these underlying populations are in constant change and interaction.

Hence, the CPS, as presented here, are not in a fixed relationship with the international standards. Rather that relationship, in the context of specific temporal, epidemiological and environmental conditions, is dynamic in terms of the prevalence and heterogeneity of the associated phenotypes. However, despite the variability in risk factors and even genetic markers of disease across populations, the biological impact (clinical or pathophysiological manifestations) on these risk factors or genetic markers for common diseases are consistent across populations (Ioannidis et al., 2004).

In summary, we propose to implement universal strategies in the study of the complexity of early human phenomena and the CPS. *Integration* across healthy, normative populations and *complexity* when it comes to pathological determinations and their possible courses of restoration with *simplification* to molecular levels. This should be a strategy for better understanding, but not an end in itself. The unfulfilled expectations of direct clinical applicability, for the treatment of specific conditions, after the presentation of the human genome is an example. The experience of the COVID-19 pandemic is another, with health services not yet prepared despite the previous alarms and the technological development of very effective vaccines, encountering large resistance to their application in developed countries while virus replication rampant in underserved regions.

In summary, CPS are clinical, pathophysiological and developmental entities with unique implications for humans. They are complex because they are caused by a combination of pregnancy-related conditions, socio-economic, lifestyle and environmental factors. In the postnatal years, the resulting offspring are characterized by aetiologically-based phenotypes that influence their health, growth and development up to adulthood. As CPS merge multi-causal aetiological factors (complex) to postnatal morbidity, growth and development (syndrome), they require a whole-organism approach to conditions previously treated as separate clinical entities, such as PTB, FGR, postnatal growth faltering, and early child developmental delay.

## Data Availability Statement

The original contributions presented in the study are included in the article/supplementary material, further inquiries can be directed to the corresponding author.

## Author Contributions

RF, MR, and JV conceptualized this manuscript with input from JH and SK. RF, MR, JV, and JVO wrote the first draft of the manuscript and other authors contributed and revised it critically for important intellectual content. All authors approved the final version for publication.

## Conflict of Interest

The authors declare that the research was conducted in the absence of any commercial or financial relationships that could be construed as a potential conflict of interest.

## Publisher’s Note

All claims expressed in this article are solely those of the authors and do not necessarily represent those of their affiliated organizations, or those of the publisher, the editors and the reviewers. Any product that may be evaluated in this article, or claim that may be made by its manufacturer, is not guaranteed or endorsed by the publisher.
